# Protection against β-amyloid-induced synaptic and memory impairments via altering β-amyloid assembly by bis(heptyl)-cognitin

**DOI:** 10.1038/srep10256

**Published:** 2015-07-21

**Authors:** Lan Chang, Wei Cui, Yong Yang, Shujun Xu, Wenhua Zhou, Hongjun Fu, Shengquan Hu, Shinghung Mak, Juwei Hu, Qin Wang, Victor Pui-Yan Ma, Tony Chung-lit Choi, Edmond Dik-lung Ma, Liang Tao, Yuanping Pang, Michael J. Rowan, Roger Anwyl, Yifan Han, Qinwen Wang

**Affiliations:** 1 Department of Physiology, Ningbo Key Laboratory of Behavioral Neuroscience, Zhejiang Provincial Key Laboratory of Pathophysiology, School of Medicine, Ningbo University. Ningbo 315211, China; 2 Department of Applied Biology and Chemistry Technology, Institute of Modern Chinese Medicine, the Hong Kong Polytechnic University, Hong Kong, China; 3 Department of Pharmacology, Zhongshan School of Medicine, Sun Yat-Sen University, Guangzhou 510275, China; 4 Department of Chemistry, Hong Kong Baptist University, Hong Kong, China; 5 Mayo Foundation for Medical Education and Research, Rochester 55906-5425, MN, USA; 6 Trinity College Institute of Neuroscience, Department of Pharmacology and Therapeutics, Department of Physiology; 7 Trinity College Institute of Neuroscience, Department of Pharmacology and Therapeutics, Trinity College, Dublin 2, Ireland

## Abstract

β-amyloid (Aβ) oligomers have been closely implicated in the pathogenesis of Alzheimer’s disease (AD). We found, for the first time, that bis(heptyl)-cognitin, a novel dimeric acetylcholinesterase (AChE) inhibitor derived from tacrine, prevented Aβ oligomers-induced inhibition of long-term potentiation (LTP) at concentrations that did not interfere with normal LTP. Bis(heptyl)-cognitin also prevented Aβ oligomers-induced synaptotoxicity in primary hippocampal neurons. In contrast, tacrine and donepezil, typical AChE inhibitors, could not prevent synaptic impairments in these models, indicating that the modification of Aβ oligomers toxicity by bis(heptyl)-cognitin might be attributed to a mechanism other than AChE inhibition. Studies by using dot blotting, immunoblotting, circular dichroism spectroscopy, and transmission electron microscopy have shown that bis(heptyl)-cognitin altered Aβ assembly via directly inhibiting Aβ oligomers formation and reducing the amount of preformed Aβ oligomers. Molecular docking analysis further suggested that bis(heptyl)-cognitin presumably interacted with the hydrophobic pockets of Aβ, which confers stabilizing powers and assembly alteration effects on Aβ. Most importantly, bis(heptyl)-cognitin significantly reduced cognitive impairments induced by intra-hippocampal infusion of Aβ oligomers in mice. These results clearly demonstrated how dimeric agents prevent Aβ oligomers-induced synaptic and memory impairments, and offered a strong support for the beneficial therapeutic effects of bis(heptyl)-cognitin in the treatment of AD.

Alzheimer’s disease (AD) is a progressive neurodegenerative disorder characterized by the loss of memory and cognitive functions associated with synaptic impairments in the brain. Recent studies have shown that synaptic impairments, including the disruption of synaptic plasticity and the loss of synapses, rather than neuronal degeneration, are synchronous with impairment of cognitive functions^1^,^2^, suggesting that synaptic impairments should be considered as the primary therapeutic target for the treatment of AD.

Accumulation of extracellular amyloid plaque is considered a pathological feature of AD. β-amyloid (Aβ) could form small soluble oligomers followed by assembly into protofibrils and fibrils via a complex, multistep-nucleated polymerization^1^. There is a much stronger relationship between cognitive status and the concentration of soluble Aβ oligomers rather than Aβ monomers or fibrils. It is widely accepted that soluble Aβ oligomers might lead to cognitive impairment even in the early stage when there is little evidence of neurodegeneration^2^. In animals studies, Aβ oligomers selectively impairs synaptic transmissions, reduces the number of synapses and inhibit synaptic plasticity^3^. These lines of evidence strongly suggest that the accumulation of soluble Aβ oligomers rather than Aβ monomers or fibrils may play central roles in the pathogenesis of AD.

Many studies have shown that Aβ assembly and the toxicity of Aβ oligomers could be manipulated by small molecules^4^,^5^. Curcumin and its derivatives were found to block Aβ oligomerization and enhance memory in Aβ-infused rats^1^,^4^. An orcein-related molecule, O4, was reported to reduce the concentration of Aβ oligomers and reverse Aβ oligomers-inhibited long-term potentiation (LTP) by accelerating the formation of amyloid fibrils^5^. Cyclohexanehexol stereoisomers, which inhibit Aβ aggregation, were shown to reduce AD pathology in a transgenic mouse model^6^. It is suggested that molecules with the property of Aβ assembly alteration might be a powerful tool for AD therapy.

Currently FDA-approved anti-AD drugs are limited to acetylcholinesterase (AChE) inhibitors and N-methyl-D-aspartate (NMDA) receptor antagonists based on the link between cholinergic dysfunction, excitotoxicity and severity of this disease^7^. AChE possesses two active sites, namely central anion site (CAS) and peripheral anion sites (PAS). Traditional AChE inhibitors including tacrine and donepezil mainly act on the CAS of AChE. Bis(heptyl)-cognitin is a novel dimeric AChE inhibitor derived from tacrine, designed to target both CAS and PAS of AChE^8^. As compared to tacrine, bis(heptyl)-cognitin showed 1000 times more potent in inhibiting rat brain AChE^8^. Our previous studies demonstrated that bis(heptyl)-cognitin possesses superior properties in memory enhancement potency in rats and also attenuates Aβ-induced neuronal apoptosis *in vitro*^9^,^10^. However, the underlying mechanisms of the action of bis(heptyl)-cognitin are largely unknown and it remains to be elucidated whether bis(heptyl)-cognitin can protect against Aβ oligomers-induced synaptic impairments. In this study, we investigated the effects of bis(heptyl)-cognitin on Aβ oligomers-induced synaptic and memory impairments in various *in vitro* and *in vivo* models. Our results suggested that bis(heptyl)-cognitin significantly attenuated Aβ oligomers-induced synaptic and memory impairments by altering Aβ assembly, possibly via directly interacting Aβ.

## Material and Methods

### Chemicals and reagents

Bis(heptyl)-cognitin was synthesized as previously described by us^11^. The purity of bis(heptyl)-cognitin was evaluated by using liquid chromatography-mass spectrometry. Bis(heptyl)-cognitin was dissolved in Milli-Q water at a concentration of 1 mM and stored frozen at −20 °C. Before being used, bis(heptyl)-cognitin was further diluted with Milli-Q water. Donepezil, tacrine, methyllycaconitine (MLA) and hexafluoroisopropanol (HFIP) were purchased from Sigma (St Louis, MO, USA). Curcumin, KT5720, MG624 and H89 were purchased from Tocris (Bristol, UK). Curcumin, donepezil, KT5720, MG624 and H89 were dissolved in dimethyl sulfoxide (DMSO) with a maximum final concentration of 0.1% (DMSO). Other chemicals were prepared in Milli-Q water. All media and supplements used for cell culture were from Invitrogen (Carlsbad, CA).

### A statement on the ethical handling of animals

All rodent experiments were conducted according to the ethical guidelines of Animal Subjects Ethics Sub-committee (ASESC), the Hong Kong Polytechnic University; and the protocol was approved by ASESC, the Hong Kong Polytechnic University (permit number: 10/15). All surgeries were performed under sodium pentobarbital anesthesia, and all efforts were made to minimize animal suffering.

### Preparation of soluble Aβ_1-42_ oligomers

Soluble Aβ_1-42_ oligomers were prepared as previously described by our laboratory with modification^10^. Synthetic Aβ_1-42_ (Bachem, Torrance, CA) was dissolved in HFIP to a concentration of 1 mM, and 100 μl aliquots dried overnight evaporated under vacuum. The dried Aβ_1-42_ was then reconstituted in 20 μl DMSO, and sonicated in a sonicating bath for 5 min. Then Aβ_1-42_ was diluted to 100 μM by using culture medium. After incubation for 48 hours at 4 °C, Aβ_1-42_ solution was centrifuged at 14000 g for 10 min at 4 °C, and the soluble Aβ_1-42_ (mainly oligomers) in the supernatant was collected and quantified by BCA assay.

### Preparation of rat hippocampal slice and electrophysiological techniques

All electrophysiological experiments were carried out on transverse slices of Wistar rat hippocampus. The brains were rapidly removed after decapitation and placed in cold oxygenated media. Slices were cut at a thickness of 350 μm using and placed in a storage container containing oxygenated medium at room temperature for 1 h. The slices were then transferred to a recording chamber for submerged slices and continuously superfused at a rate of 5-6 ml/min at 30-32 °C. The control media contained mM of: NaCl, 120; KCl, 2.5; NaH_2_P0_4_, 1.25; NaHCO_3_, 26; MgSO_4_, 2.0; CaCl_2_, 2.0; D-glucose 10. All solutions contained picrotoxin (100 μM) to block GABA_A_-mediated responses.

The slices were transferred to a recording chamber, where they were held submerged between two nylon nets and maintained at 30-32 °C. The chamber consisted of a circular well of a low volume and was perfused constantly at a rate of 4-5 ml/min. All experiments were carried out in the dentate gyrus, with presynaptic stimulation applied to the medial perforant pathway of the dentate gyrus using a bipolar insulated tungsten wire electrode, and field excitatory postsynaptic potentials (fEPSPs) recorded at a control test frequency of 0.033 Hz from the middle one-third of the molecular layer of the dentate gyrus with a glass microelectrode.

In experiments involving application of Aβ_1-42_ oligomers, Aβ_1-42_ oligomers were perfused for 45 min prior to high frequency stimulation (HFS). In experiments involving AChE inhibitors, AChE inhibitors or vehicle were perfused over the slices for 60 min prior to HFS. Control and experimental levels of LTP were measured on slices prepared from the same hippocampus. Experiments that involved α7 type nicotinic acetylcholine receptor (α7nAChR) and cAMP-dependent protein kinase (PKA) inhibitors, and experiments that involved the effect of inhibitors alone and the effects of inhibitors applied together with Aβ_1-42_ oligomers were carried out on slices of the same hippocampus. All values were normalized to this baseline and all values of LTP reported here were calculated as changes in fEPSP amplitude measured 60 min after tetanic stimulation.

### Preparation of primary rat hippocampal neurons

Primary cultured hippocampal neurons were obtained from 18-day-old Sprague-Dawley (SD) rat embryos as previously described by us with minor modification^12^. Briefly, the hippocampus was dissected and incubated with 0.25% trypsin at 37 °C. Cells were then mechanically dissociated by using a Pasteur pipette with a fire-narrowed tip in culture medium and plated at a density of 2 × 10^5^ cells/ml on 35-mm culture dishes pre-coated with poly-L-lysine (10 μg/ml).Cells were maintained in neurobasal/B27 medium containing 0.5 mM glutamine, 100 units/ml penicillin, 100 μg/ml streptomycin at 37 °C. Half-changes of medium were done twice weekly.

### Immunocytochemistry and image acquisition

All steps were performed at room temperature as described previously^13^ unless otherwise stated. For synapse quantification, primary hippocampal neurons were fixed for 20 min in 4% paraformaldehyde, 10% sucrose, 15 μg/ml Hoechst-33342 in PBS, then washed twice with PBS. Cultures were incubated in blocking buffer (10% goat serum, 1% BSA, 0.1% Triton X-100 in PBS) for 1 h, followed by addition of rabbit anti-synapsin-1 antibody (Calbiochem, Darmstadt, Germany), in blocking buffer for 1 h. Cells were subsequently washed three times with washing buffer (1% BSA, 0.1% Triton X-100 in PBS) before addition of mouse anti-βIII-tubulin antibody (Cell signaling, Beverly, MA) in blocking buffer for 1 h. Following three additional washes, Alexafluor-488 anti-mouse and Alexafluor-568 anti-rabbit secondary antibodies, diluted 1:300 in blocking buffer, were added simultaneously for 1 h. Cells were finally washed three times by PBS before analysis.

### Image acquisition and analysis

Image acquisition was carried out according to previous publications with modification^13^,^14^. Briefly, images were acquired by a Nikon ECLIPSE Ti-U microscope (Nikon Instruments Inc, Melville, NY) at × 400 magnifications. All images in this study were acquired as 12-bit TIFF images. Optimal settings were achieved empirically as we had changed those parameters until images with clear and distinctive signals were obtained. Image analysis was carried out using ImageJ Software according to previous publications with modification^13^,^14^. Briefly, ultraviolet excitation and emission wavelengths were used to obtain images of nuclei labeled with Hoechst-33342, which allowed identification of the correct focal plane for further image acquisition. Second and third excitation wavelengths were used to illuminate neurites and synapses labeled with Alexa-568 and -488 secondary antibodies, respectively. Neuronal cell bodies were identified as βIII-tubulin positive objects. Neurites were identified as βIII-tubulin positive structures, and analyzed by a NeuriteTracer program^15^. Briefly, image pairs were opened as neuronal and nuclear stacks. Then, images were pre-processed to equalize the illumination with the stack and reduce imaging artifacts. Following pre-processing, images were thresholded and speckles were moved. The thresholded neuronal marker images were skeletonized and measured. Synapsin I integrated immunofluorescence intensity at each pixel across the images by using ImageJ^16^. Cell bodies were digitally removed from the images so that synapsin I immunostaining on βIII-tubulin positive neurites was quantified. All of the above selection criteria were user-defined and all subsequent image analysis used the same criteria.

### Immunoblotting analysis

The inhibition of Aβ_1-42_ oligomers formation was performed as previously described with modification^1^. Briefly, synthetic Aβ_1-42_ was dissolved in HFIP to 1 mg/ml, evaporated under vacuum, and reconstituted in DMSO to 5 mM. Aβ_1-42_ was diluted to 50 μM by using phenol red-free DMEM medium and incubated with various agents for 24 h at 4 °C. The Aβ_1-42_ solution was centrifuged at 14000 g for 10 min, and the soluble oligomeric Aβ_1-42_ in the supernatant was mixed with an equal part of Tricine sample buffer without reducing agents. Samples were electrophoresed and transferred. Membranes were boiled for 10 min, and blocked for 1 hour at room temperature, probed with rabbit anti-Aβ_1-17_ antibody 6E10 (Sigma, 1:1000) followed by goat anti-rabbit horseradish peroxidase, and developed with an enhanced chemiluminescence plus kit (Amersham Bioscience, Aylesbury, UK).

### Dot blotting analysis

Dot blotting analysis was performed as previously described^17^. Briefly, nitrocellulose membrane was divided into 1.5 cm grid lines and 2 μl of each sample was spotted onto the membrane with air-dry. The membrane was blocked in 10% milk TBST solution overnight. Then the membrane was incubated with anti-oligomer antibody A11 (Invitrogen, 1:1000) or anti-Aβ_1-17_ antibody 6E10 (Sigma, 1:1000) with gentle shaking for 1 h. After three washes with TBST, the membrane was incubated with secondary antibodies for 1 h, developed with an enhanced chemiluminescence plus kit and quantified by using ImageJ.

### Circular dichroism spectroscopy

Circular dichroism (CD) spectroscopy was performed as previously described^18^. Briefly, synthetic Aβ_1-42_ was dissolved in HFIP to 1 mg/ml, evaporated under vacuum, and reconstituted in DMSO to 5 mM. Then Aβ_1-42_ was diluted to 20 μM by using 10 mM sodium phosphate (pH = 7.4), and incubated with or without 1 μM bis(heptyl)-cognitin. CD spectra were acquired at 15 min, 6 h, 1d and 2d after incubation at 37 °C. CD measurements were made by removing a 200 μl aliquot from the reaction mixture, adding the aliquot to a 1 mM path length CD cuvette and acquiring spectra in a J-805spectropolarimeter (JASCO, Tokyo, Japan). Following temperature equilibration, spectra were recorded from ~195-240 nm at 0.2 nm resolution with a scan rate of 100 nm/min. Raw data were manipulated by smoothing and subtraction of buffer spectra according to the manufacturer’s instructions.

### Transmission electron microscopy

Assay of Aβ_1-42_ oligomers formation using Transmission electron microscopy (TEM) images was performed as previously described^5^. Briefly, synthetic Aβ_1-42_ was dissolved in HFIP to 1 mg/ml, evaporated under vacuum, and reconstituted in DMSO to 5 mM. Then Aβ_1-42_ was diluted to 20 μM by using 10 mM sodium phosphate (pH = 7.4), and incubated with 1 μM bis(heptyl)-cognitin. The TEM samples were prepared by placing 2 μl of the pre-incubated solution on a carbon-coated grid. The samples were stained with 1% uranylacetate and then placed on a clean paper for removing excess staining solution. The grids were thoroughly examined using JEM-2011transmission electron microscope (JEOL, Tokyo, Japan).

### Molecular docking analysis

The solution NMR structure of Aβ_1-42_ assemblies (PDB: 2BEG)^19^ was taken from PDB and prepared for molecular modeling using the Internal Coordinate Mechanics method (ICM-pro 3.6-1d, MolSoft L.L.C., La Jolla, CA)^20^. According to the ICM method, the molecular system was described by using internal coordinates as variables. Energy calculations were based on the ECEPP/3 force field with a distance-dependent dielectric constant. The biased probability Monte Carlo minimization procedure was used for global energy optimization.

ICM docking was performed to find out the most favourable orientation. The resulting trajectories of the complex between the small molecules and Aβ_1-42_ were energy minimized, and the interaction energies, which are expressed in kJ/mol, were computed. The compound was docked three times and the minimum of the three scores was used.

### Aβ_1-42_ oligomers administration and drug treatment

Aβ_1-42_ oligomers administration was performed as previously described with modification^21^. Briefly, male ICR mice (20-26 g) anesthetized with chloral hydrate were positioned in a Narishige stereotaxic instrument, and 1 μl Aβ_1-42_ oligomers (1 μg/μl) were injected into the bilateral hippocampi. Sham control mice were infused with the vehicle. Both bis(heptyl)-cognitin and tacrine were dissolved in saline.

Administration of bis(heptyl)-cognitin (0.1 or 0.2 mg/kg, *i.p.*), tacrine (1 or 2 mg/kg, *i.p.*), or vehicle (saline) once per day for 12 consecutive days, began on the fifth day of the infusion of Aβ_1-42_ oligomers.

### Morris water maze task

The water maze apparatus consisted of a circular pool 110 cm in diameter, filled with water at 23 ± 2 °C to cover a platform. The platform always resided in the center of southwest quadrant except on the last day. Each mouse’s swimming was monitored by a video camera linked to a computer-based image analyzer. Learning performance was tested for 5 consecutive days beginning at 12th day after the infusion of Aβ_1-42_ oligomers. Each mouse was trained to find the platform, with two trials a day. In each trial, the time required to escape onto the hidden platform was recorded. On day 5 of training, a probe trial was made by removing the platform and allowing the mice to swim for 90 s in search of the platform. Swimming time and distance in each of the four quadrants in the pool were calculated as percentages of the totals. A persistent preference for the quadrant previously occupied by the platform was taken to indicate that the mice had acquired and remembered the spatial task.

### Data analysis and statistics

The data are expressed as the means ± SEM. Statistical significance was determined by ANOVA with Dunnett’s test in the case of multiple comparisons with control or Tukey’s test. Differences were accepted as significant at *p* < 0.05.

## Results

### Bis(heptyl)-cognitin enhances HFS-induced LTP

**Figure 1:**
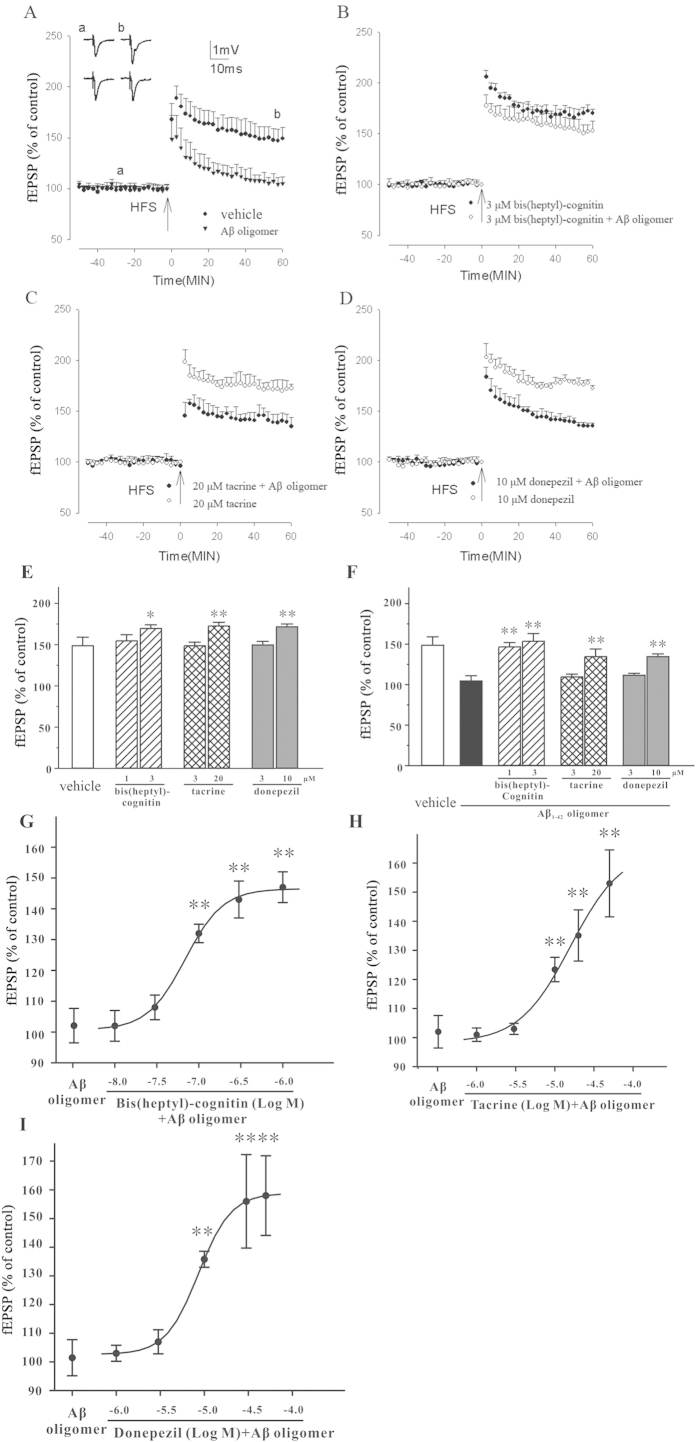
Bis(heptyl)-cognitin, but not other AChE inhibitors, enhances HFS-induced LTP, and prevents Aβ oligomers-induced inhibition of LTP at low concentrations. (**A**) Aβ_1-42_ oligomers inhibit HFS-induced LTP. The graph shows the induction of LTP by vehicle (filled circles) or by 0.5 μM Aβ_1-42_ oligomers, perfused for 45 min prior to HFS (filled triangle). (**B**-**D**) Bis(heptyl)-cognitin (3 μM), tacrine (20 μM) and donepezil (10 μM) enhance HFS-induced LTP, and prevent Aβ_1-42_ oligomers-induced inhibition of LTP. Bis(heptyl)-cognitin, tacrine and donepezil were perfused over the slices for 60 min prior to HFS. (**E**) Bis(heptyl)-cognitin (3 μM), tacrine (20 μM) and donepezil (10 μM) enhance HFS-induced LTP. (**F**) Bis(heptyl)-cognitin (3 μM), tacrine (20 μM) and donepezil (10 μM) prevent Aβ_1-42_ oligomers-induced inhibition of LTP. Bis(heptyl)-cognitin, tacrine and donepezil were perfused over the slices for 60 min prior to HFS. After 15 min, Aβ_1-42_ oligomers were perfused. (**G**-**I**) The concentration-dependent effects of bis(heptyl)-cognitin (**G**), tacrine (**H**) or donepezil (**I**) on Aβ_1-42_ oligomers-induced inhibition of LTP. Data represent means ± SEM (n = 5). ^*^p < 0.05 and ^**^p < 0.01 *vs.* vehicle group in (**E**); and ^**^p < 0.01 *vs.* Aβ_1-42_ oligomers group in (**F**-**I**) (ANOVA and Tukey’s test).

Consistent with our previous studies^22^,^23^, HFS induced NMDA receptor-dependent LTP. The average LTP measurement was 149 ± 10% at 60 min post-HFS (n = 6; Fig. 1A,E). In order to study the effects of bis(heptyl)-cognitin on LTP, we first chose a concentration of bis(heptyl)-cognitin that had been reported to be effective against neuronal apoptosis (1 μM)^24^. Two typical AChE inhibitors, tacrine and donepezil, were used in concentrations that were chosen by their AChE inhibition and therapeutic ranges^7^,^25^. AChE inhibitors were applied 60 min before application of HFS and they remained present throughout the experiments. Although other AChE inhibitors at high concentrations significantly enhanced LTP following HFS compared to vehicle control (20 μM tacrine, n = 5, *p* < 0.01 compared to vehicle LTP; 10 μM donepezil, n = 5, *p* < 0.01 compared to vehicle LTP; Fig. 1E), they failed to increase HFS-induced LTP at low concentrations (3 μM tacrine, n = 5, *p* > 0.05 compared to vehicle LTP; 3 μM donepezil, n = 5, *p* > 0.05 compared to vehicle LTP; Fig. 1E). In com*p*arison to vehicle LTP, 3 μM bis(heptyl)-cognitin enhanced HFS-induced LTP (n = 5, *p* < 0.05, Fig. 1E).

### Bis(heptyl)-cognitin, but not other AChE inhibitors, prevents Aβ_1-42_ oligomers-induced inhibition of LTP at low concentrations

Acute superfusion of hippocampal slices with Aβ_1-42_ oligomers (500 nM) for 45 min significantly reduced LTP induction by HFS (105 ± 6%, n = 8, *p* < 0.01 compared to vehicle LTP; Fig. 1A,F). The baseline amplitudes were not significantly different between vehicle and Aβ_1-42_ oligomers exposed slices. We further examined the effects of AChE inhibitors on Aβ_1-42_ oligomers-induced inhibition of LTP at concentrations that would not enhance HFS-induced LTP, *i.e.* bis(heptyl)-cognitin at 1 μM, tacrine at 3 μM, and donepezil at 3 μM. AChE inhibitors were perfused over the slices for 60 min prior to HFS. After 15 min, Aβ_1-42_ oligomers were perfused. Bis(heptyl)-cognitin, but not other AChE inhibitors, completely prevented Aβ_1-42_ oligomers-induced inhibition of LTP at low concentrations (Fig. 1F).

To further examine the threshold concentration of bis(heptyl)-cognitin for the prevention of Aβ_1-42_ oligomers-induced inhibition of LTP, serial concentrations of AChE inhibitors were used. Our results showed that bis(heptyl)-cognitin prevented Aβ_1-42_ oligomers-induced inhibition of LTP in a concentration-dependent manner with the threshold concentration at approximately 0.1 μM (Fig. 1G). Tacrine and donepezil prevented Aβ_1-42_ oligomers-inhibited inhibition of LTP with the threshold concentrations at 10 μM and 10 μM, respectively (Fig. 1H,I).

### Blockage of α7nAChR could not abolish the prevention of Aβ_1-42_ oligomers-induced inhibition of LTP caused by bis(heptyl)-cognitin

**Figure 2:**
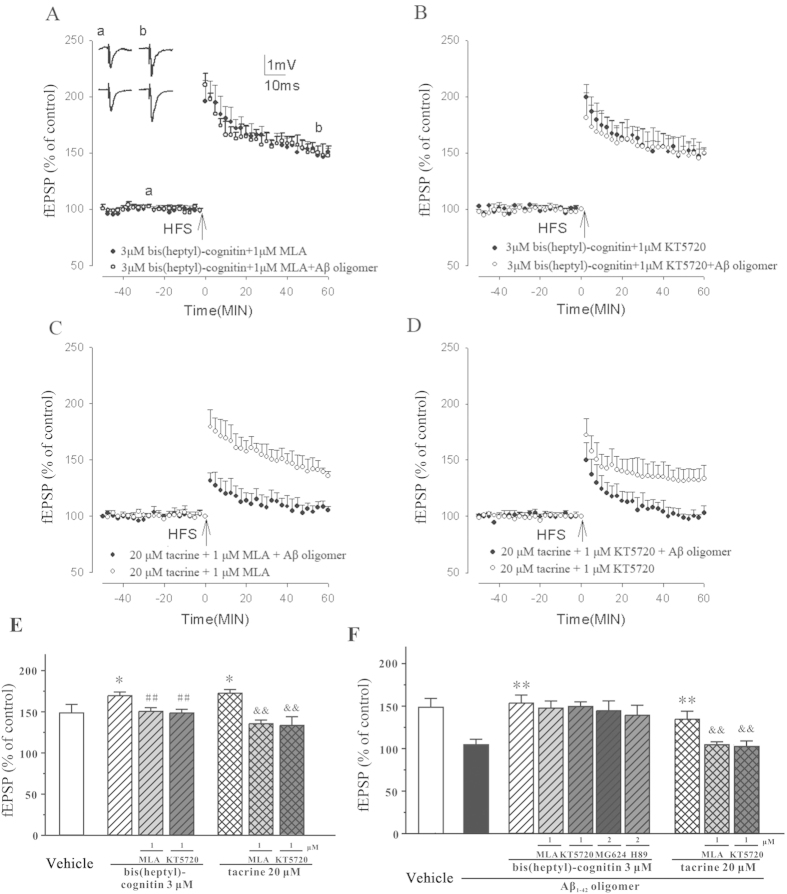
Blockage of α7nAChR could not abolish the prevention of Aβ_1-42_ oligomers-induced inhibition of LTP by bis(heptyl)-cognitin. (**A**-**D**) MLA and KT5720 block the enhancement of HFS-induced LTP, but not the prevention of Aβ_1-42_ oligomers-induced inhibition of LTP by bis(heptyl)-cognitin. Bis(heptyl)-cognitin, tacrine and kinase inhibitors were perfused over the slices for 60 min prior to HFS. After 15 min, Aβ_1-42_ oligomers were perfused. (**E**) α7nAChR mediates the enhancement of HFS-induced LTP caused by either bis(heptyl)-cognitin or tacrine. (**F**) Blockage of α7nAChR could not abolish the prevention of Aβ_1-42_ oligomers-induced inhibition of LTP by bis(heptyl)-cognitin. Data represent means ± SEM (n = 5). ^*^p < 0.05 *vs.* vehicle; ^##^*p* < 0.01 or ^&&^*p* < 0.01 *vs.* bis(heptyl)-cognitin or tacrine group, respectively in (**E**); ^**^p < 0.01 *vs.* Aβ_1-42_ oligomers group; ^&&^*p* < 0.01 *vs.* tacrine plus Aβ_1-42_ oligomers groups in (**F**) (ANOVA and Tukey’s test).

AChE inhibitors could enhance HFS-induced LTP and prevent Aβ-mediated LTP inhibition via potentiating α7nAChR and activating downstream PKA pathway^26^,^27^,^28^. In our study, MLA, a specific α7nAChR inhibitor, and KT5720, a specific inhibitor of PKA, could block the enhancement of HFS-induced LTP and the prevention of Aβ_1-42_ oligomers-induced inhibition of LTP by tacrine (Fig. 2E,F). Interestingly, although MLA and KT5720 reduced the increase of HFS-induced LTP caused by 3 μM bis(heptyl)-cognitin, neither of these agents affected the protection of bis(heptyl)-cognitin on Aβ_1-42_ oligomers-induced inhibition of LTP (Fig. 2E,F). Moreover, MG624 (a specific α7nAChR inhibitor) and H89 (a specific PKA inhibitor) could not abolish the protection of bis(heptyl)-cognitin on Aβ_1-42_ oligomers-induced inhibition of LTP (Fig. 2F). To date, none of these inhibitors altered baseline neurotransmission or the induction of LTP by HFS in our experiments.

### Bis(heptyl)-cognitin, but not tacrine, prevents Aβ_1-42_ oligomers-induced synaptotoxicity in primary hippocampal neurons

**Figure 3:**
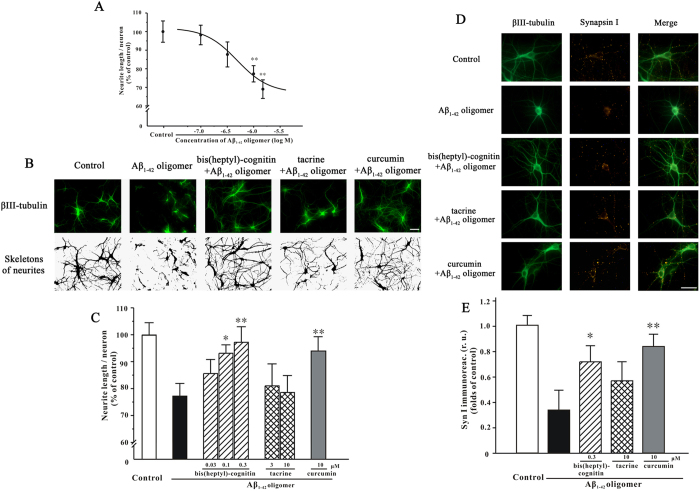
Bis(heptyl)-cognitin, but not tacrine, prevents Aβ_1-42_ oligomers-induced synaptotoxicity in primary mature hippocampal neurons. (**A**) Aβ_1-42_ oligomers reduce neurite length in hippocampal neurons. After DIV14, hippocampal neurons were treated for 4 d with various concentrations of Aβ_1-42_ oligomers prior to fixation and analysis for the length of βIII-tubulin positive neurites by using NeuriteTracer program. (**B**) Hippocampal neurons were pre-incubated with 0.3 μM bis(heptyl)-cognitin, 10 μM tacrine or 10 μM curcumin, and exposed to 1 μM Aβ_1-42_ oligomers 2 h later. Four days after Aβ_1-42_ oligomers challenge, neurons were fixed. Upper: Neuronal cultures were stained with anti-βIII-tubulin antibody. Lower: βIII-tubulin positive neurites were digitally identified and skeletonized for quantification by NeuriteTracer program (scale bar: 10 μM). (**C**) Hippocampal neurons were pre-incubated with various treatments as indicated and exposed to 1 μM Aβ_1-42_ oligomers 2 h later. Four days after Aβ_1-42_ oligomers challenge, neurons were fixed and analyzed for the length of βIII-tubulin positive neurites by using NeuriteTracer program. (**D**) Hippocampal neurons were pre-incubated with 0.3 μM bis(heptyl)-cognitin, 10 μM tacrine or 10 μM curcumin and exposed to 1 μM Aβ_1-42_ oligomers 2 h later. Four days after Aβ_1-42_ oligomers challenge, neurons were fixed and labeled with βIII-tubulin and synapsin I antibodies (scale bar: 10 μM). (**E**) Synapsin I integrated immunofluorescence intensity was evaluated by using ImageJ. r.u.: relative unit. Data represent means ± SEM (5 images were analyzed in each group), ^**^p < 0.01 *vs.* control in (**A**); ^*^p < 0.05 and ^**^p < 0.01 *vs.* Aβ_1-42_ oligomers group in (**C**) and (**E**) (ANOVA and Dunnett’s test).

It is well established that Aβ_1-42_ oligomers not only inhibit synaptic plasticity, but also induce synaptotoxicity in hippocampal neurons^13^. To further study the effects of bis(heptyl)-cognitin on the prevention of synaptic impairments, an immunostaining-based quantitative study was used. At 14 day *in vitro* (DIV), primary mature hippocampal neurons were exposed to Aβ_1-42_ oligomers for 4d. Aβ_1-42_ oligomers (0.1-1.5 μM) caused a concentration-dependent reduction in the length of βIII-tubulin positive neurites with a threshold concentration of 1 μM (Fig. 3A).

Curcumin was reported to prevent Aβ_1-42_ oligomers formation *in vitro* and reduce Aβ_1-42_ toxicity in hippocampal neurons^1^,^29^. We confirmed that pretreatment with 10 μM curcumin for 2 h prevented neurite length reduction induced by Aβ_1-42_ oligomers (*p* < 0.01, Fig. 3C). The effects of bis(heptyl)-cognitin and tacrine were also examined. Bis(heptyl)-cognitin (0.1-0.3 μM) prevented Aβ_1-42_ oligomers-induced reduction of neurite length in a concentration-dependent manner (Fig. 3C). However, tacrine even at 10 μM could not prevent the reduction of neurite length caused by Aβ_1-42_ oligomers (Fig. 3C).

Synapsin I integrated immunofluorescence intensity was also evaluated in our study. Immunofluorescence intensity of synapsin I was significantly decreased in hippocampal neurons after 4d of treatment with Aβ_1-42_ oligomers (Fig. 3D,E). This decrease was prevented by 2 h pretreatment with bis(heptyl)-cognitin (0.3 μM) and curcumin (10 μM), but not tacrine (10 μM) (Fig. 3E).

### Bis(heptyl)-cognitin inhibits the formation of Aβ_1-42_ oligomers and reduces the amount of pre-formed Aβ_1-42_ oligomers

**Figure 4:**
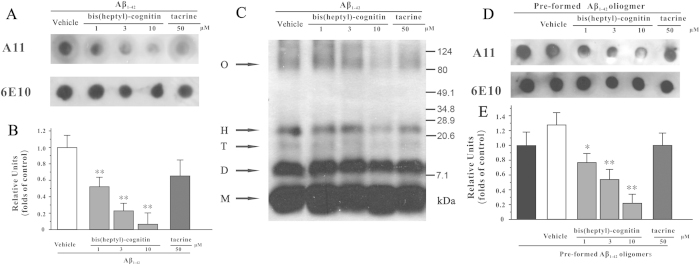
Bis(heptyl)-cognitin inhibits Aβ_1-42_ oligomers formation, and reduces the amount of preformed Aβ_1-42_ oligomers. (**A**) Bis(heptyl)-cognitin inhibits Aβ_1-42_ oligomers formation. 50 μM disassembled (HFIP-pretreated) Aβ_1-42_ was incubated with various agents as indicated for 48 hours at 4 °C. Aβ_1-42_ solution was then centrifuged at 14000 g for 10 min, the supernatant was spotted onto the membrane. Then the membrane was immunoblotted with anti-oligomer antibody (A11) or anti-Aβ_1-17_ antibody (6E10). (**B**) Statistic analysis of relative density of A11 dots in each treatment group. Data were expressed as the ratio to OD values of the corresponding controls. Data, expressed as percentage of control, were the mean ± SEM of three separate experiments; ^**^p < 0.01 *versus* Aβ_1-42_ group (ANOVA and Dunnett’s test). (**C**) Bis(heptyl)-cognitin reduces the amount of Aβ_1-42_ oligomers. 50 μM disassembled Aβ_1-42_ was incubated with various agents as indicated for 48 hours at 4 °C. Aβ_1-42_ solution was then centrifuged at 14000 g for 10 min. The supernatant was electrophoresed at 100 V on a 15% Tris-Tricine SDS gel and probed with anti-Aβ_1-17_ antibody (6E10). M, monomer; D, dimer; T, trimer; H, hexamer; O, medium-size oligomer. Assemblies ranging from dimers to medium-size oligomer were recognized as Aβ oligomers. (**D**) Bis(heptyl)-cognitin reduces the amount of preformed Aβ_1-42_ oligomers. 50 μM disassembled Aβ_1-42_ was incubated for 48 hours at 4 °C, and then treated with various agents as indicated for 2 hours at 4 °C. Aβ_1-42_ solution was then centrifuged at 14000 g for 10 min, the supernatant was spotted onto the membrane. Then the membrane was immunoblotted with anti-oligomer antibody (A11) or anti-Aβ_1-17_ antibody (6E10). (**E**) Statistic analysis of relative density of A11 dots in each treatment group. Data were expressed as the ratio to OD values of the corresponding controls. Data, expressed as percentage of control, were the mean ± SEM of three separate experiments; *p < 0.05 and ^**^p < 0.01 *versus* pre-formed Aβ_1-42_ oligomers group (ANOVA and Dunnett’s test).

In order to test whether the anti-Aβ oligomers-induced synaptic impairments effects of bis(heptyl)-cognitin were related to the alteration of Aβ assembly, Aβ_1-42_ oligomerization assay and dot blotting analysis were used. We dissolved 50 μM Aβ_1-42_ in HFIP to reconstitute a “seedless” monomer and then incubated it with bis(heptyl)-cognitin in an oligomer formation protocol^1^. The resulting oligomers-enriched supernatants were analyzed by immunoblotting and dot blotting analysis with an oligomer-specific antibody A11 and a general Aβ antibody 6E10. It was found that bis(heptyl)-cognitin (1-10 μM) substantially inhibited the formation of Aβ_1-42_ oligomers (Fig. 4A,B).

Assemblies ranging from dimers to 24-mers (molecule weight at 8-100 kDa) were widely accepted as Aβ_1-42_ oligomers^30^,^31^,^32^. We then investigated the ability of bis(heptyl)-cognitin to reduce different forms of Aβ_1-42_ oligomers. When 50 μM “seedless” Aβ_1-42_ was allowed to assemble without bis(heptyl)-cognitin, three bands between 7.1 and 28.9 kDa representing the dimer, trimer and hexamer, and a medium-sized oligomer smear at 80-124 kDa were present (Fig. 4C). As the concentration of bis(heptyl)-cognitin increased from 1 to 10 μM, the amount of trimer, hexamer and medium-sized oligomers decreased considerably (Fig. 4C).

To further examine whether bis(heptyl)-cognitin could affect pre-formed Aβ oligomers, we prepared Aβ oligomers in an oligomer formation protocol, and then treated with bis(heptyl)-cognitin. The resulting oligomer-enriched supernatants were analyzed by dot blotting analysis. Our results showed that bis(heptyl)-cognitin but not tacrine substantially reduced the amount of pre-formed Aβ oligomers (Fig. 4D,E).

### Bis(heptyl)-cognitin alters Aβ assembly

**Figure 5:**
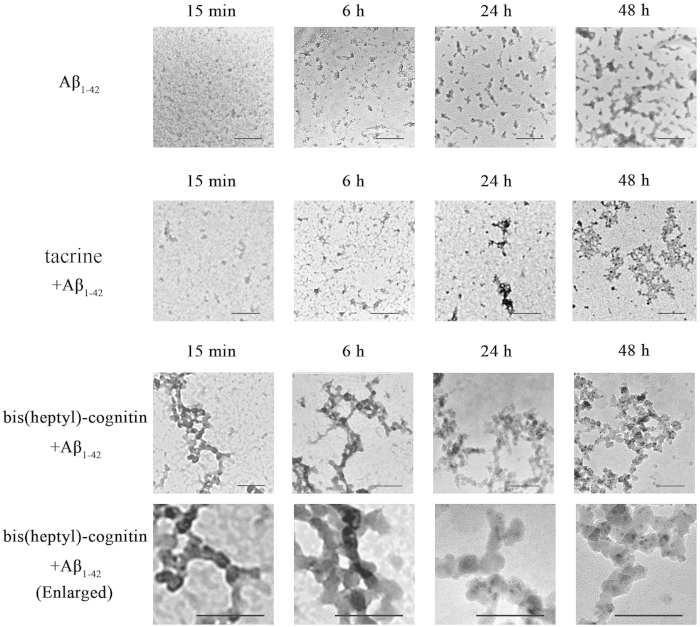
Bis(heptyl)-cognitin changes transitions in secondary structure during Aβ assembly. Changes in secondary structure indicated by far-UV CD during assembly of 20 μM Aβ_1-42_ alone or with 1 μM bis(heptyl)-cognitin or 10 μM tacrine. Aliquots of reactions (200 μl) were transferred at various times to a CD cuvette, and spectra were recorded. Representative spectra are shown for Aβ_1-42_ samples taken at the indicated times alone (**A**) in the presence of 1 μM bis(heptyl)-cognitin (**B**) or 10 μM tacrine (**C**). The spectra presented at each time were representative of those obtained during each of the five independent experiments.

To further examine whether bis(heptyl)-cognitin modified the secondary structure of Aβ during Aβ assembly, we undertook CD studies. Aβ_1-42_, incubated alone, produced the initial spectra characteristic (Fig. 5A). Aβ_1-42_ displayed substantial secondary structural changes between 15 min and 2d, results that were consistent with previously reported pro-β-sheet transitions associated with monomer-protofibril-fibril assembly. When 20 μM Aβ_1-42_ was incubated with 1 μM bis(heptyl)-cognitin, no such transitions were observed (Fig. 5B), suggesting that bis(heptyl)-cognitin modified the secondary structural changes during Aβ assembly. Tacrine hardly changed the initial spectral characteristic of Aβ during Aβ assembly (Fig. 5C).

**Figure 6:**
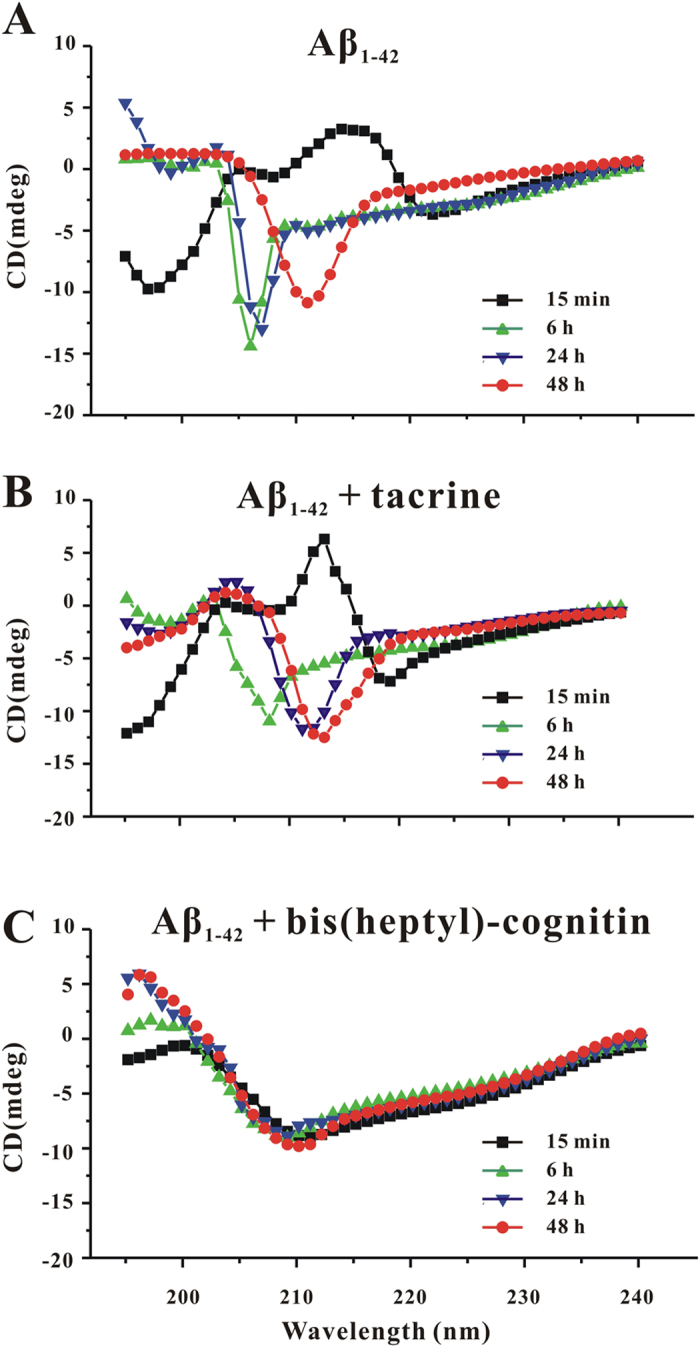
Electron micrographs of Aβ_1-42_ (20 μM) assembled alone or with 1 μM bis(heptyl)-cognitin or with 10 μM tacrine at various time points as indicated. The high power images are enlarged from low power images. Scale bar = 100 nm.

To determine the morphology of Aβ_1-42_ assemblies in the samples, TEM was used. Globular Aβ_1-42_ oligomers with diameters of about 10-20 nm were detected predominantly in the untreated Aβ_1-42_ samples (Fig. 6). In contrast, mostly large chain-like assemblies were observed in the bis(heptyl)-cognitin-treated samples (Fig. 6). These chain-like assemblies were detected even at 15 min after bis(heptyl)-cognitin and Aβ_1-42_ co-incubation. Globular oligomers were mainly observed in the tacrine-treated samples (Fig. 6).

### Bis(heptyl)-cognitin binds favorably to the hydrophobic clefts of Aβ assemblies

**Figure 7:**
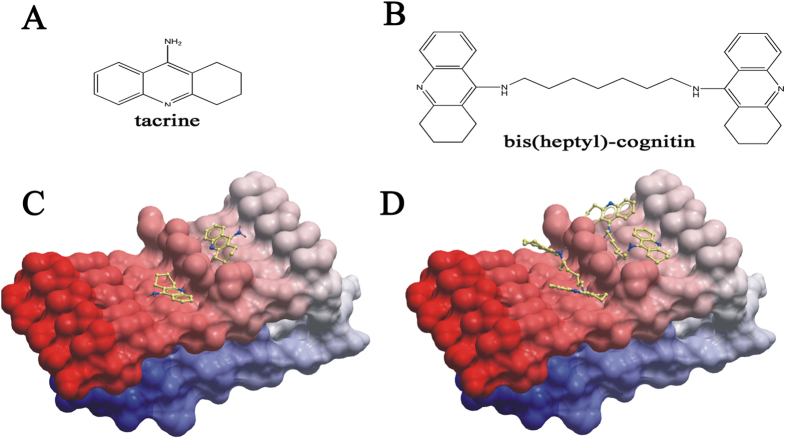
The potential interaction between bis(heptyl)-cognitin and Aβ. (**A**) Chemical structure of tacrine. (**B**) Chemical structure of bis(heptyl)-cognitin. Low-energy binding conformations of bis(heptyl)-cognitin (**C**) or tacrine (**D**) bound to the surface of Aβ assemblies (Gly33-Met35 and Met35-Gly37) generated by molecular docking. The small molecule is depicted as a ball-and-stick model showing carbon (yellow), nitrogen (blue), and hydrogen (dark grey) atoms. The Aβ assemblies are shown as skin representation.

To elucidate the mechanism underlying the alteration of Aβ assembly by bis(heptyl)-cognitin, we performed molecular docking analysis by using ICM-pro 3.6-1d molecular docking algorithm. Since no co-crystal structure of inhibitor with Aβ was available, the solution NMR structure of Aβ assemblies (PDB: 2BEG) was taken from PDB and used for docking analysis. In our model, no detectable hydrogen bonds between small molecules (bis(heptyl)-cognitin and tacrine) and Aβ assemblies were recorded. The interactions between small molecules and Aβ assemblies were mainly governed by hydrophobic interactions. Bis(heptyl)-cognitin was predicted to bind favorably to the hydrophobic clefts formed by Gly33-Met35 and Met35-Gly37 on the surface of Aβ_1-42_ (Fig. 7). Surprisingly, bis(heptyl)-cognitin situated longitudinally to Aβ assemblies where its two tacrine subunits tied up four to five units of Aβ_1-42_ molecules that are linked by the heptyl linkers, while the related monomer tacrine was only predicted to interact with two Aβ molecules (Fig. 7). Collectively, our results suggested that bis(heptyl)-cognitin presumably interacted with the hydrophobic pockets (Gly33-Met35 and Met35-Gly37) through multiple hydrophobic interactions which confers stabilizing powers and assembly alteration effects on Aβ.

### Bis(heptyl)-cognitin reduces cognitive impairments after infusion of Aβ oligomers in mice

**Figure 8:**
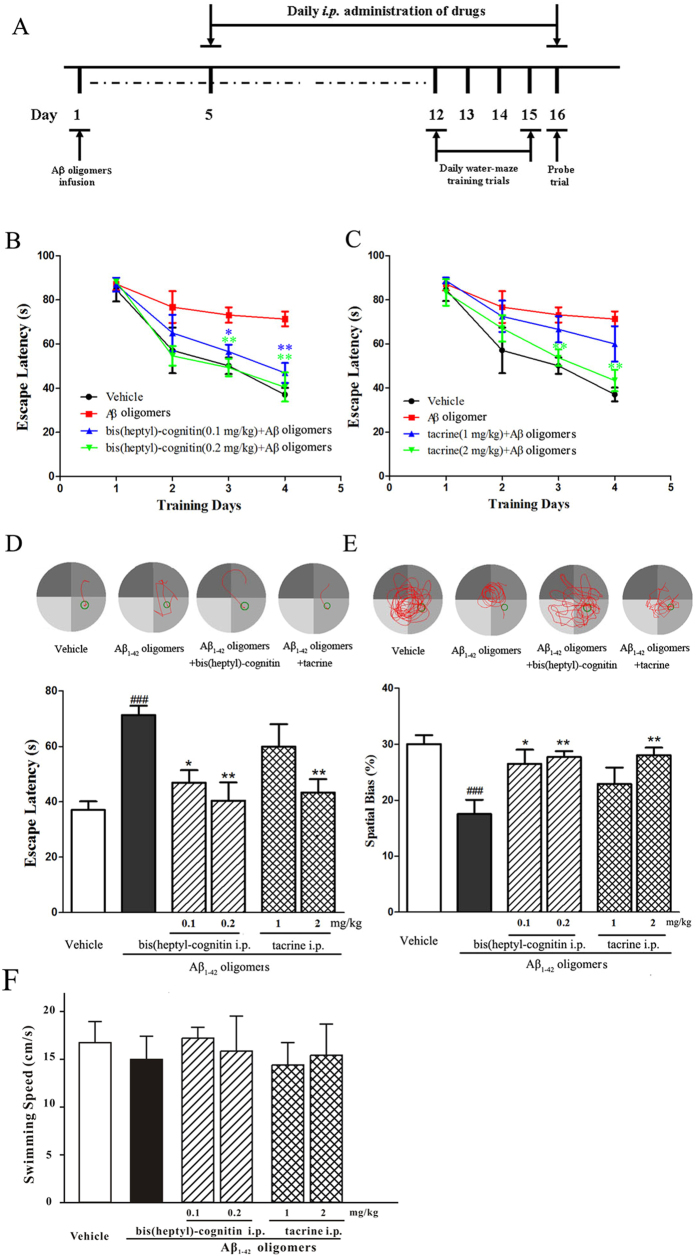
Bis(heptyl)-cognitin prevents the memory deficits induced by intra-hippocampal infusion of Aβ_1–42_ oligomers in mice (n = 6 for each group). (**A**) The schedules of animal experiments. (**B**,**C**) Mean latencies to escape from the water onto the hidden platform. Each mouse was subjected to two trials per day for 4 consecutive days. (**D**) Upper: typical swimming-tracking path of vehicle control, Aβ_1-42_ oligomers-treated mice, bis(heptyl)-cognitin 0.2 mg/kg plus Aβ_1-42_ oligomers-treated mice, and tacrine 2 mg/kg plus Aβ_1-42_ oligomers-treated mice on the fourth training day. Lower: mean latencies to escape from the water onto the hidden platform on the fourth training day. (**E**) Upper: typical swimming-tracking path of of vehicle control, Aβ_1-42_ oligomers-treated mice, bis(heptyl)-cognitin 0.2 mg/kg plus Aβ_1-42_ oligomers-treated mice, and tacrine 2 mg/kg plus Aβ_1-42_ oligomers-treated mice in the probe trial. Lower: the swimming distance in the target quadrant (in which the platform had been placed during the training phase) in the probe trial. (**F**) Average swimming speed in the probe trial. Data represent means ± SEM. ^*###*^*p* < 0.001 *vs.* vehicle group; ^*^p < 0.05 and ^**^p < 0.01 *vs.* Aβ_1-42_ oligomers-treated group in (**B**-**E**) (Tukey’s test).

Previous studies have shown that Aβ_1-42_ oligomers lead to the learning and memory impairments in mice^33^,^34^. In our study, Morris water maze was used to evaluate spatial memory of mice with administration of Aβ_1-42_ oligomers by intra-hippocampal infusion (Fig. 8A). In the acquisition trials, typical swimming paths on the fourth training day and quantitative escape latencies indicated that mice treated with Aβ_1-42_ oligomers took longer time to find the platform than did the vehicle-treated mice (*p* < 0.01, Fig. 8D). This prolongation of latency was significantly shortened by bis(heptyl)-cognitin at the concentrations of 0.1 and 0.2 mg/kg, and tacrine at the concentrations of 2 mg/kg (*p* < 0.01, Fig. 8D). In the probe trials, the swimming distance in the quadrant that had held the hidden platform was used to estimate performance. The swimming distance in the probe quadrant is longer in groups of mice treated with Aβ_1-42_ oligomers plus bis(heptyl)-cognitin (0.1, 0.2 mg/kg) and Aβ_1-42_ oligomers plus tacrine (2 mg/kg) than the group of mice treated with Aβ_1-42_ oligomers alone (*p* < 0.01, Fig. 8E). Neither Aβ_1-42_ oligomers nor drugs altered swimming speed (Fig. 8F).

## Discussion

Bis(heptyl)-cognitin was first reported as a potent AChE inhibitor^8^. Previous studies have reported that synaptic transmission in the hippocampus is enhanced by the activation of α7nAChR and its downstream PKA pathway^35^. Inhibition of AChE could lead to the accumulation of acetylcholine in the synaptic cleft, and result in the activation of α7nAChR and the enhancement of normal LTP. We have shown that 1) bis(heptyl)-cognitin and other AChE inhibitors increase HFS-induced LTP in hippocampal slice; and 2) the enhancement of LTP by bis(heptyl)-cognitin and other AChE inhibitors could be abolished by α7nAChR antagonists and PKA inhibitors. These results suggested that bis(heptyl)-cognitin, as well as other AChE inhibitors, increased HFS-induced LTP via activating α7nAChR/PKA pathway.

We have further compared the prevention of bis(heptyl)-cognitin on Aβ oligomers-induced inhibition of LTP with tacrine and donepezil. All these AChE inhibitors prevented Aβ oligomers-induced inhibition of LTP but with different threshold concentrations. It is published that α7nAChR agonists could prevent the inhibition of LTP by Aβ_1-42_ oligomers, which could be abolished by α7nAChR antagonists, suggesting that the activation of α7nAChR prevents Aβ_1-42_ oligomers-mediated inhibition of LTP^36^. In our study, both α7nAChR antagonists and PKA inhibitors abolished the prevention of Aβ oligomers-induced inhibition of LTP by tacrine, suggesting that these AChE inhibitors prevent Aβ oligomers-induced inhibition of LTP via the activation of α7nAChR/PKA pathway. These results are in accordance with previous studies showing that typical AChE inhibitors prevent Aβ-impaired LTP via potentiating α7nAChR/PKA pathway^26^,^36^.

Interestingly, bis(heptyl)-cognitin, but not other AChE inhibitors, prevented Aβ oligomers-induced inhibition of LTP at concentrations that did not interfere with normal LTP. The protection of bis(heptyl)-cognitin on Aβ oligomers-induced inhibition of LTP could not be inhibited by α7nAChR antagonists or PKA inhibitors. These bis(heptyl)-cognitin may act on target(s) other than α7nAChR. The modification of Aβ oligomers-induced inhibition of LTP by bis(heptyl)-cognitin might be attributed to a mechanism different from that of typical AChE inhibitors.

Several studies have shown that agents which alter Aβ assembly could prevent Aβ-mediated LTP inhibition in hippocampal slices^5^,^37^. Moreover, curcumin (1 μM), a known blocker of Aβ oligomerization, significantly prevented Aβ oligomers-induced inhibition LTP in our model (data not shown), suggesting that bis(heptyl)-cognitin might prevent Aβ oligomers-induced inhibition of LTP via altering Aβ assembly.

Apart from the inhibition of LTP, other aspects of synaptic impairments including the decrease in neurite length and the loss of synapses, were also reported to be highly correlated with cognitive impairments in AD^38^. By using a quantitative assay based on synapsin I and βIII-tubulin co-immunostaining, we investigated the effects of Aβ oligomers on synaptotoxicity in primary mature hippocampal neurons^13^,^39^. Treatments of Aβ oligomers for four days displayed a concentration-dependent decrease in neurite length in hippocampal neurons with a minimal concentration of 1 μM. However, shorter exposure (less than 2d) of 1 μM Aβ oligomers did not exhibit any significant alternation in our study (data not shown). In our study, acute application of Aβ oligomers resulted in LTP inhibition in the hippocampal slice but not detectable synaptotoxicity in our primary culture system. Our previous studies have shown that acute Aβ oligomers-induced LTP impairment was mediated by the activation of MAPK pathways, and the stimulation of inducible nitric oxide synthase and superoxide^40^,^41^. However, these acute effects produced by Aβ oligomers may not be sufficient to cause detectable synaptotoxicity in our primary culture system. Chronic treatment of Aβ oligomers could induce long-term oxidative stress, tau protein hyper-phosphorylation, insulin signaling impairments *etc*, leading to detectable synaptotoxicity^42^,^43^,^44^. It has been reported that agents that alter Aβ assembly can reduce Aβ oligomers-induced synaptotoxicity^45^. We found that curcumin at 10 μM almost completely prevented Aβ oligomers-induced synaptotoxicity. Using fluorescent microscopy and a powerful image analysis software, it was determined that bis(heptyl)-cognitin significantly prevented Aβ oligomers-induced synaptotoxicity. However, tacrine did not produce such effects. These results also match their effects on the prevention of Aβ oligomers-induced inhibition of LTP.

How could bis(heptyl)-cognitin prevent synaptic impairments induced by Aβ oligomers? We have shown that bis(heptyl)-cognitin significantly reduced the formation of Aβ oligomers and decreased the amount of pre-formed Aβ oligomers. Western blotting analysis further demonstrated that bis(heptyl)-cognitin preferably reduced the formation of Aβ oligomers. Previous studies have shown that Aβ assembly is a highly complex process that involves the sequential formation of different forms of amyloid assemblies, such as monomers, oligomers, protofibrils and mature fibrils^46^. Aβ monomers could assemble into unstructured oligomers, which convert over time into protofibrils and fibrils with a β-sheet-rich structure^47^. Our CD study showed that bis(heptyl)-cognitin froze secondary structure transitions during Aβ assembly. Our TEM data further suggested that Aβ monomers formed large chain-like assemblies but not small globular oligomers in the presence of bis(heptyl)-cognitin.

Tacrine, the monomeric form of bis(heptyl)-cognitin, even at a very high concentration, hardly altered Aβ assembly, suggesting that the dimeric form of bis(heptyl)-cognitin might be critical for altering Aβ assembly. A previous study has revealed a high free energy barrier between Aβ oligomers and large Aβ assemblies^48^. Although large Aβ assemblies have much lower free energy than Aβ oligomers, the assembly from Aβ oligomers into large Aβ assemblies involves crossing over this high free energy barrier, implying that Aβ oligomers are quite stable and could not be easily assembled into large Aβ assemblies^48^.

Our molecular docking results suggested that tacrine might bind to Aβ molecule in large Aβ assemblies. Bis(heptyl)-cognitin situated longitudinally to large Aβ assemblies where its two tacrine subunits tied up four to five units of Aβ molecules. Therefore, the interaction between bis(heptyl)-cognitin and large Aβ assemblies might be stronger than that between tacrine and large Aβ assemblies, indicating that bis(heptyl)-cognitin might be more potent than tacrine to stabilize large Aβ assemblies. These results also suggested that bis(heptyl)-cognitin might decrease the high free energy barrier between other Aβ forms and large Aβ assemblies, and facilitate assembly from Aβ oligomers into large Aβ assemblies. Interestingly, although the concentrations of Aβ and bis(heptyl)-cognitin used in various *in vitro* experiments are different, the molecular ratios of bis(heptyl)-cognitin to Aβ in these studies are similar, being close to 1:5, which further support our molecular docking prediction that bis(heptyl)-cognitin might promote Aβ assembly from Aβ oligomers into large Aβ assemblies with one bis(heptyl)-cognitin molecule tied up four to five Aβ molecules.

Previously, many these compounds with symmetric structures are known to inhibit Aβ oligomerization. We conjured that the symmetric structure of these compounds, including bis(heptyl)-cognitin in particular, might facilitate their binding to the regular Aβ assemblies, leading to the inhibition of Aβ oligomers. However, the detailed mechanisms are under investigated in our laboratories.

Finally, we have investigated the effects of bis(heptyl)-cognitin in mice with intra-hippocampal infusion of Aβ_1-42_ oligomers. Evidenced by the decrease in escape latency during the hidden platform sessions and the increase in swimming distance in the target quadrant as compared with the Aβ_1-42_ oligomers-treated group, bis(heptyl)-cognitin could significantly ameliorate the impairments of learning and memory following Aβ_1-42_ oligomers administration. Although tacrine could also reduce learning and memory impairments in this model, bis(heptyl)-cognitin has shown much higher efficacy (0.1 mg/kg *vs.* 2 mg/kg) and higher potency than tacrine in reducing these impairments.

In conclusion, we have demonstrated that bis(heptyl)-cognitin attenuated Aβ oligomers-induced synaptic and memory impairments. Our previous study has shown that bis(heptyl)-cognitin is a potent AChE inhibitor^8^. AD is a multifaceted neurodegenerative disorder. Therapeutic pharmacological approaches with one-drug-one-target are limited in their abilities to treat such a complex disease. One-molecule-multi-target drugs might provide greater therapeutic efficacy by concurrently targeting different sites in the brain of AD patients. AChE inhibitors have been proven to be effective to stabilize the symptoms of AD. Aβ oligomers are considered the main neurotoxin for synaptic dysfunctions and memory impairment in the AD progress. Therefore, it is likely that multi-functional molecules, such as bis(heptyl)-cognitin, which target AChE and Aβ oligomers simultaneously might not only minimize cholinergic dysfunction but also reverse the cognitive impairments in AD. In our study, we have demonstrated that bis(heptyl)-cognitin, a novel AChE inhibitor derived from tacrine, could reduce the neurotoxicity of Aβ oligomers, possibly via altering Aβ assembly, suggesting that bis(heptyl)-cognitin might be effective in the treatment of AD.

## Additional Information

**How to cite this article**: Chang, L. *et al*. Protection against ß-amyloid-induced synaptic and memory impairments via altering ß-amyloid assembly by bis(heptyl)-cognitin. *Sci. Rep.* 5, 10256 doi: 10.1038/srep10256 (2015).
